# Implementation of hepatitis B vaccine in high-risk young adults with waning immunity

**DOI:** 10.1371/journal.pone.0202637

**Published:** 2018-08-20

**Authors:** Nawarat Posuwan, Arnond Vorayingyong, Vorapol Jaroonvanichkul, Rujipat Wasitthankasem, Nasamon Wanlapakorn, Sompong Vongpunsawad, Yong Poovorawan

**Affiliations:** 1 Center of Excellence in Clinical Virology, Faculty of Medicine, Chulalongkorn University, Bangkok, Thailand; 2 Academic Administration, Faculty of Medicine, Chulalongkorn University, Bangkok, Thailand; University of Cincinnati College of Medicine, UNITED STATES

## Abstract

Universal hepatitis B (HB) vaccination among Thai newborns was initiated in 1992. The first dose of the monovalent HB vaccine was given at birth, then at months 2 and 6 simultaneously with the diphtheria-tetanus-pertussis whole-cell (DTPw) vaccine. In 2008, Thailand replaced the monovalent HB vaccine at months 2 and 6 with a combined DTP-HB given at months 2, 4, and 6, with an added monovalent HB vaccine at month 1 for infants whose mothers were HBV carriers. Despite this rigorous HB vaccination schedule, vaccinated infants who are now adolescents do not possess a protective level of anti-HB surface antigen (anti-HBs) (≥10 mIU/ml). Thus, many young adults may be rendered susceptible to HB infection. Our objective was to determine how HB booster vaccination may benefit high-risk adolescents. We evaluated the serological records of a cohort of medical students (n = 291), which showed that 271 students (93.1%) possessed anti-HBs less than the accepted protective level (<10 mIU/ml) and subsequently received the HB vaccine booster prior to medical school enrollment. We then examined the anti-HB surface antibody (anti-HBs) in 216 individuals six weeks after they were immunized. We found that 61%, 88%, and 94% of individuals with pre-booster anti-HBs of <1 mIU/ml, 1-<3 mIU/ml, and 3-<10 mIU/ml achieved protective anti-HBs, respectively. Post-booster geometric mean titers were 305, 513, and 1,929 mIU/ml in these groups and correlated with pre-booster anti-HBs titers. These data suggest that medical students with known anti-HBs <1 mIU/ml will benefit from 3 doses of HB vaccine at 0, 1, and 6 months. Students with anti-HBs 1-<10 mIU/ml would benefit from an HB vaccine booster without further anti-HBs evaluation.

## Introduction

The World Health Organization (WHO) estimates that 3.5% of the world population, or approximately 257 million individuals are infected with hepatitis B virus (HBV) [1]. In Southeast Asia alone, the prevalence of HBV infection is ~2% and affects approximately 39 million people. HBV infection frequently occurs through vertical (mother-to-infant) transmission [2]. Untreated chronic infection may eventually progress to end-stage liver disease, cirrhosis, and hepatocellular carcinoma [1].

Beginning in 1988, Thailand initiated a pilot program to reduce vertical HBV transmission through monovalent HB vaccination among newborns in two provinces (Chiang Mai and Chon Buri). In 1990, vaccination coverage expanded to 12 provinces. In 1992, universal monovalent HB vaccine was administered to all Thai newborns, five years before the WHO recommendation for global HB vaccination [3]. The vaccine was administered at birth, then at 2 and 6 months of age. For convenience, the second and third doses were provided simultaneously with diphtheria-tetanus-pertussis whole-cell (DTPw) vaccine [4, 5].

In 1994, the use of a combined DTPw-HB vaccine was initiated in Chiang Rai province. Monovalent HB vaccine was given at birth, followed by DTPw-HB vaccine at 2, 4, and 6 months of age (totaling 4 doses of HB vaccine) [6]. This vaccination schedule was expanded to 12 provinces (in 2005), 24 provinces (in 2006), 27 provinces (in 2007), and finally nationwide (in 2008). However, among infants born to HBV carrier mothers, the delayed vaccination of the second HB vaccine at month 2 instead of month 1 after birth was associated with an increased risk of vertical HBV transmission in the absence of prophylactic HB IgG at birth [7]. Consequently, infants born to HBV carrier mothers receive an additional monovalent HB vaccine at 1 month of age beginning in 2009.

Presently, the majority of Thai children and young adults born after the initiation of the universal HBV immunization program have low HBV infection rates as evaluated by HBV surface antigen (HBsAg) or anti-HBV core (anti-HBc) antibody [8]. The overall prevalence of HBV carriers has decreased dramatically among individuals born before (4.5%) compared to after (0.6%) the implementation of the universal HB vaccination program. Consequently, most HBV carriers are now primarily older Thai adults.

Our previous study showed that 79.1% of children <5 years of age possessed protective anti-HBs titers (≥10 mIU/ml). However, a decrease in anti-HBs (<10 mIU/ml) begins in adolescence. For example, only 16.9% of individuals between 11 and 20 years of age demonstrated anti-HBs ≥ 10 mIU/ml [8]. Thus, young adults with relatively low anti-HBs may be susceptible to HBV infection later in life. Medical students in the hospital setting risk occupational exposure to HBV through percutaneous injuries or mucosal contact with HBV-infected patients. Thus, the current recommendation for first-year medical students at Chulalongkorn University requires the screening of three markers of HBV, which are HBsAg, anti-HBs, and anti-HBc. For students with anti-HBs level <10 mIU/ml, they will receive three doses of HB vaccination. However, current Thai first-year medical students were born under universal HBV immunization program and most received 3–4 HB vaccines in their first years of life. We hypothesized that they do not require the three-dose HB vaccine series, but there is currently no data regarding how many doses of HB vaccination are needed in Thai high-risk young adults with waning anti-HBs immunity.

Our objective is to determine the anti-HBs response after one dose HBV booster among medical students whose anti-HBs at pre-booster were <10 mIU/ml and to make an evidence-based recommendation regarding a booster HB vaccination strategy for this population.

## Materials and methods

### Study cohort

This study was approved by the Institutional Review Board of the Faculty of Medicine, Chulalongkorn University (IRB number 363/59). All of the first-year medical students (n = 291) enrolled at the Faculty of Medicine at Chulalongkorn University were between 16 and 20 years of age. They were born after the inclusion of the HB vaccine into the universal vaccination (EPI) program. All first-year medical students underwent a school-mandated physical exam and health screening by the Faculty of Medicine, which included HB evaluation for HBsAg, anti-HBc, and anti-HBs. Since 271 individuals (93.1%) possessed anti-HBs <10 mIU/ml, the medical school provided HB vaccine (Engerix B, GlaxoSmithKline Biologicals, Rixensart, Belgium) booster for these individuals. This study began 6 weeks thereafter and followed 216 medical students (79.7%) who consented in having the serological evaluation of anti-HBs titers post-booster. Written informed consents were obtained from the students and/or parents. Proof of previous HB vaccination was not available. Based on the national vaccine coverage data for newborns, we assumed that approximately 94% of the individuals in this cohort had received three-dose HB vaccine within the first year of life.

### Laboratory methods

Anti-HBs was evaluated by an automated enzyme-linked immunosorbent assay performed in the ARCHITECT system (Abbott, Wiesbaden, Germany). The cut-off value for anti-HBs seropositivity was >1 mIU/ml, and seroprotection was defined as anti-HBs ≥10 mIU/ml.

### Data analysis

Pre-existing anti-HBs levels in individuals before the administration of the HB vaccine booster were categorized into 3 groups: <1, 1-<3 and 3-<10 mIU/ml. Sensitivity was calculated from the post-booster anti-HBs levels ≥10 mIU/ml based on the different pre-booster anti-HBs levels. Post-booster anti-HBs levels were categorized into 3 groups: <10, 10-<100, and ≥100 mIU/ml. The geometric mean titer (GMT) was calculated from the anti-HBs titer >1 mIU/ml by multiplying individuals’ anti-HBs levels and taking the n^th^ root of the product (where n is the number of observations). The GMT was represented logarithmically (log_10_ scale).

## Results

**Fig 1:**
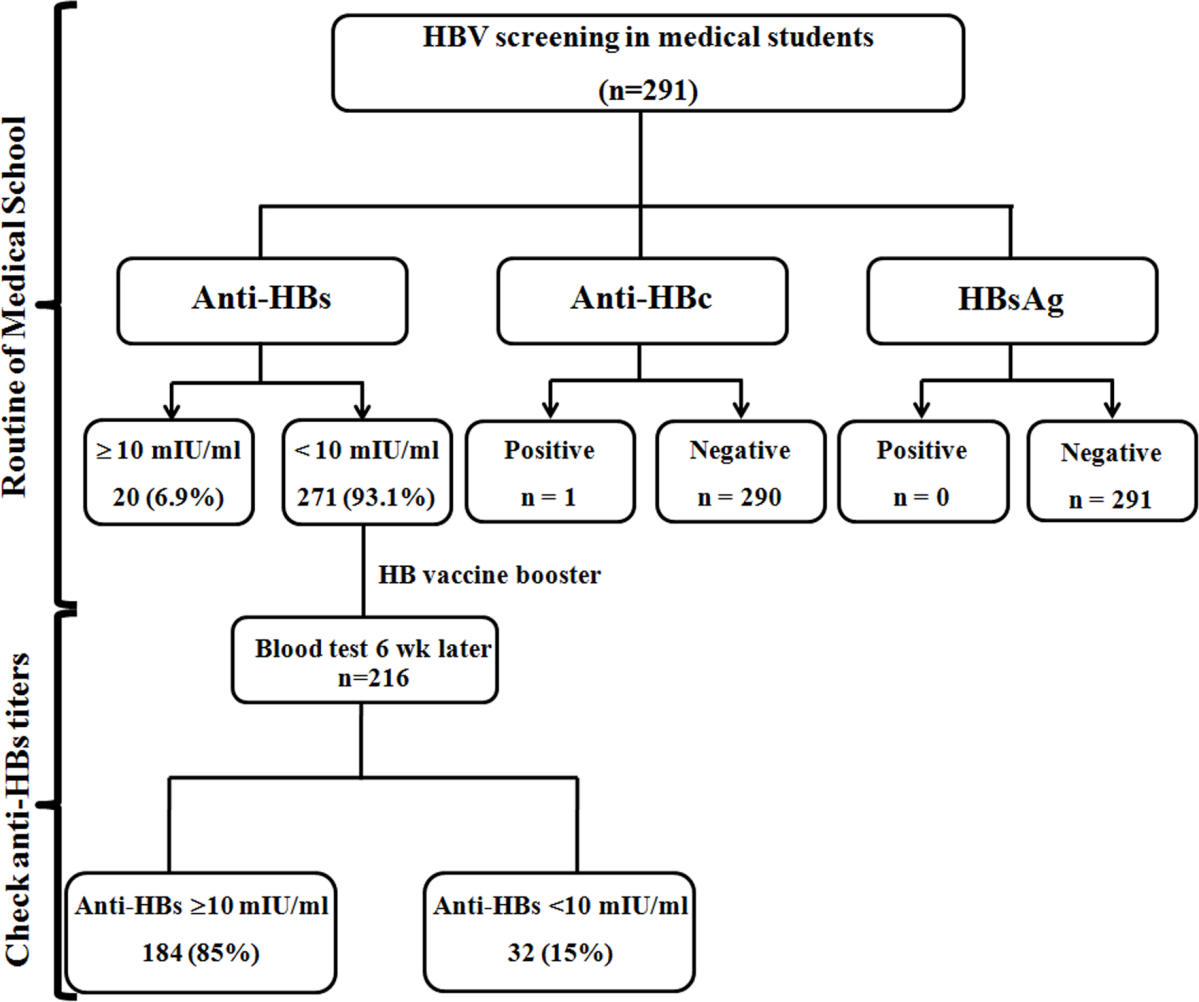
The diagram for hepatitis B serosurvey in a cohort of medical students.

Based on the school record of the initial health screening, none of the students were HBsAg-positive (Table 1). One individual had anti-HBc and anti-HBs, suggesting a previous HBV infection. Only 6.9% (20/291) of the individuals possessed anti-HBs titers ≥10 mIU/ml (GMT 79.6±4 mIU/ml). The majority (93%, 271/291) had anti-HBs of <10 mIU/ml (GMT 2.3±1.8 mIU/ml). Therefore, school record showed that 271 individuals received an HB vaccine booster (Fig 1).

**Table 1:** Characteristics of all medical students and those with post-booster HB vaccination.

Characteristics		Initial screening	Eligible for follow-up	Post-booster
		N = 291	N = 271	N = 216
Males		157 (54%)	145 (54%)	112 (52%)
Mean age (years)		17.8 ± 0.7	17.8 ± 0.6	17.8 ± 0.6
HBsAg	Positive	0	0	0
	Negative	291 (100%)	271 (%)	216 (100%)
Anti-HBc	Positive with anti-HBs	1 (0.3%)	0	0
	Positive without anti-HBs	0	0	0
	Negative	290 (99.7%)	271 (100%)	216 (100%)
Anti-HBs	<10 mIU/mL	271 (93.1%)	271 (100%)	216 (100%)
	GMT ± SD	2.3 ± 1.8	2.3 ± 1.8	2.3 ± 1.8
	≥10 mIU/mL	20 (6.9%)	-	-
	GMT ± SD	79.6 ± 4	-	-

SD denotes standard deviation

GMT denotes geometric mean titers

**Fig 2:**
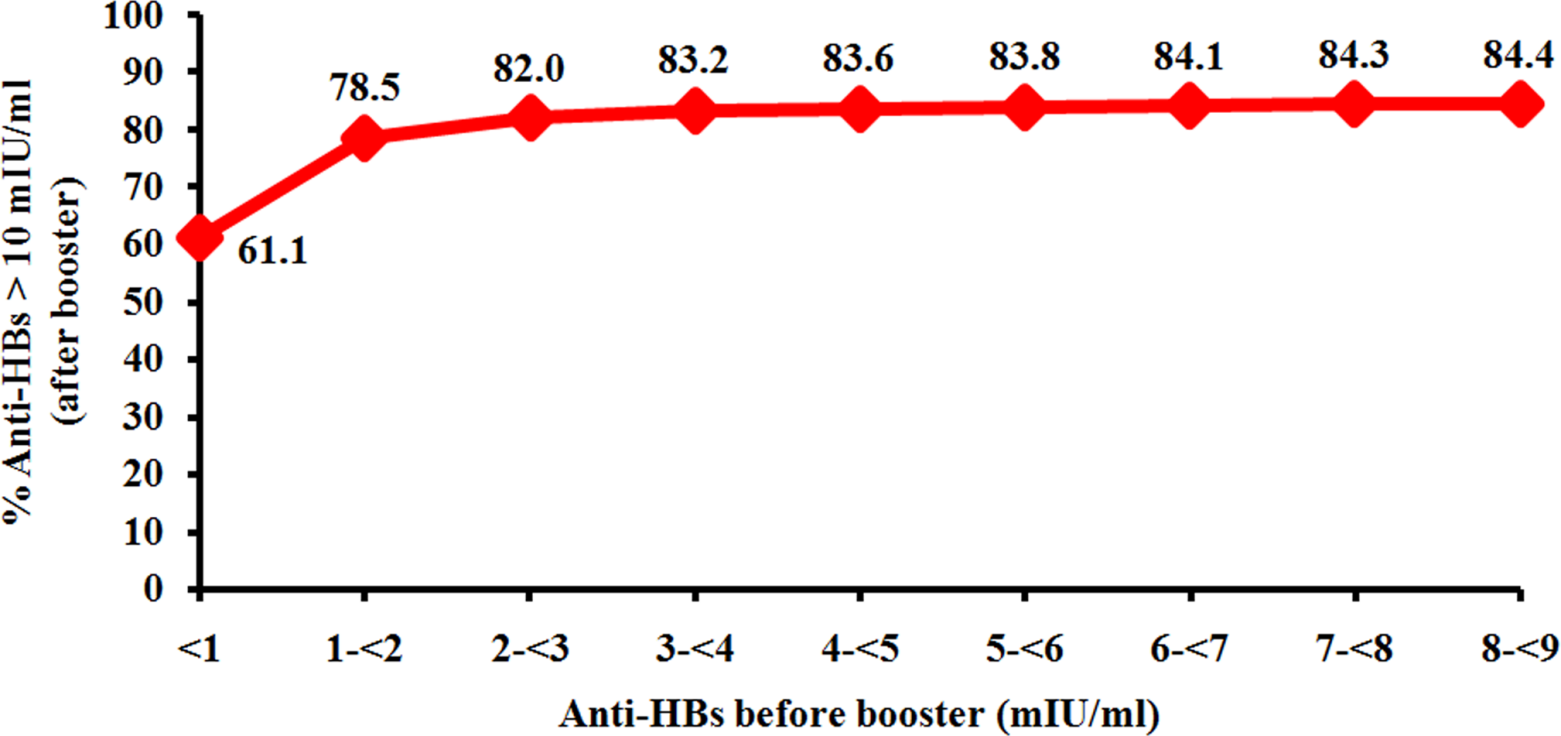
Percentage of individuals with pre-existing anti-HBs <10 mIU/ml who demonstrated protective anti-HBs status (>10 mIU/ml) post-booster.

**Fig 3:**
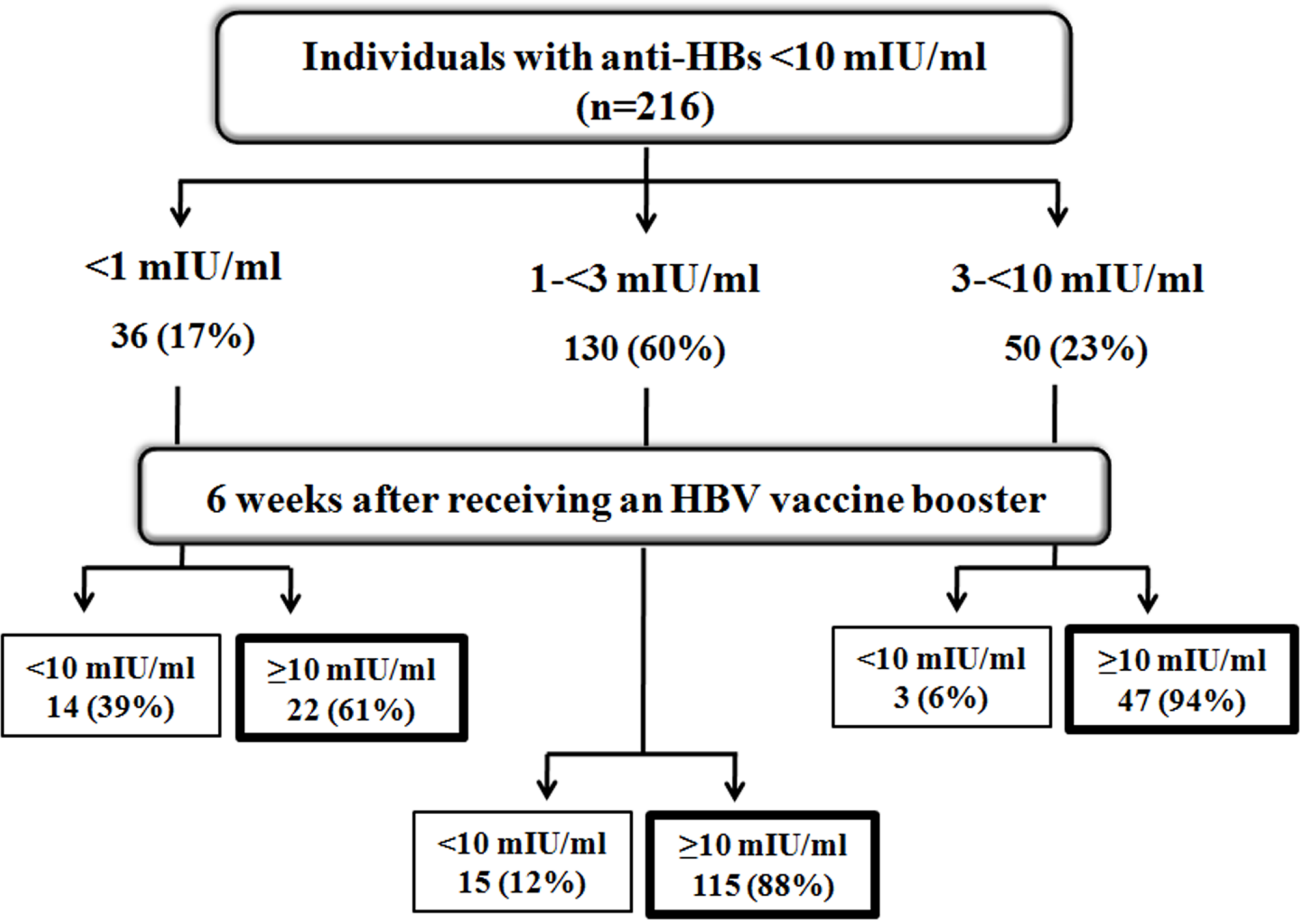
Anti-HBs levels before and after HB vaccine booster. Individuals with insufficient protective anti-HBs (<10 mIU/ml) classified into 3 groups (<1 mIU/ml, 1-<3 mIU/ml, and 3–10 mIU/ml) and had received a HB booster. Anti-HBs were evaluated 6 weeks post-booster.

Post-booster anti-HBs titers were evaluated in 216 individuals who participated in the study. Overall, 85% (184/216) demonstrated increased anti-HBs ≥10 mIU/ml, although anti-HBs in some individuals remained <10 mIU/ml (Fig 1). Since post-booster anti-HBs levels appeared to plateau for individuals with pre-booster anti-HBs of ≥3 mIU/ml (Fig 2), we stratified pre-booster anti-HBs levels into 3 groups to facilitate analysis of the data (<1 mIU/ml, 1-<3 mIU/ml and 3-<10 mIU/ml) (Fig 3). There were 36 individuals with pre-booster anti-HBs of <1 mIU/ml (17%, 36/216), 14 of whom (39%, 14/36) showed post-booster anti-HBs titer <10 mIU/ml. Among 130 individuals with pre-booster anti-HBs of 1-<3 mIU/ml, 12% (15/130) remained at <10 mIU/ml post-booster. Only 6% (3/50) of the individuals with pre-booster anti-HBs 3-<10 mIU/ml were unresponsive to the HB vaccine booster. Taken together, higher pre-booster anti-HBs titers positively correlated with the likelihood of attaining post-booster anti-HBs ≥10 mIU/ml. Thus, the achievement of HB booster in pre-booster anti-HBs of <1 mIU/ml was 61.1% (95% CI: 40.3–81.9), while pre-booster anti-HBs of 1-<3 mIU/ml was 88.5% (95% CI: 82.5–94.4) and pre-booster anti-HBs of 3-<10 mIU/ml was 94% (95% CI: 87.1–100).

**Fig 4:**
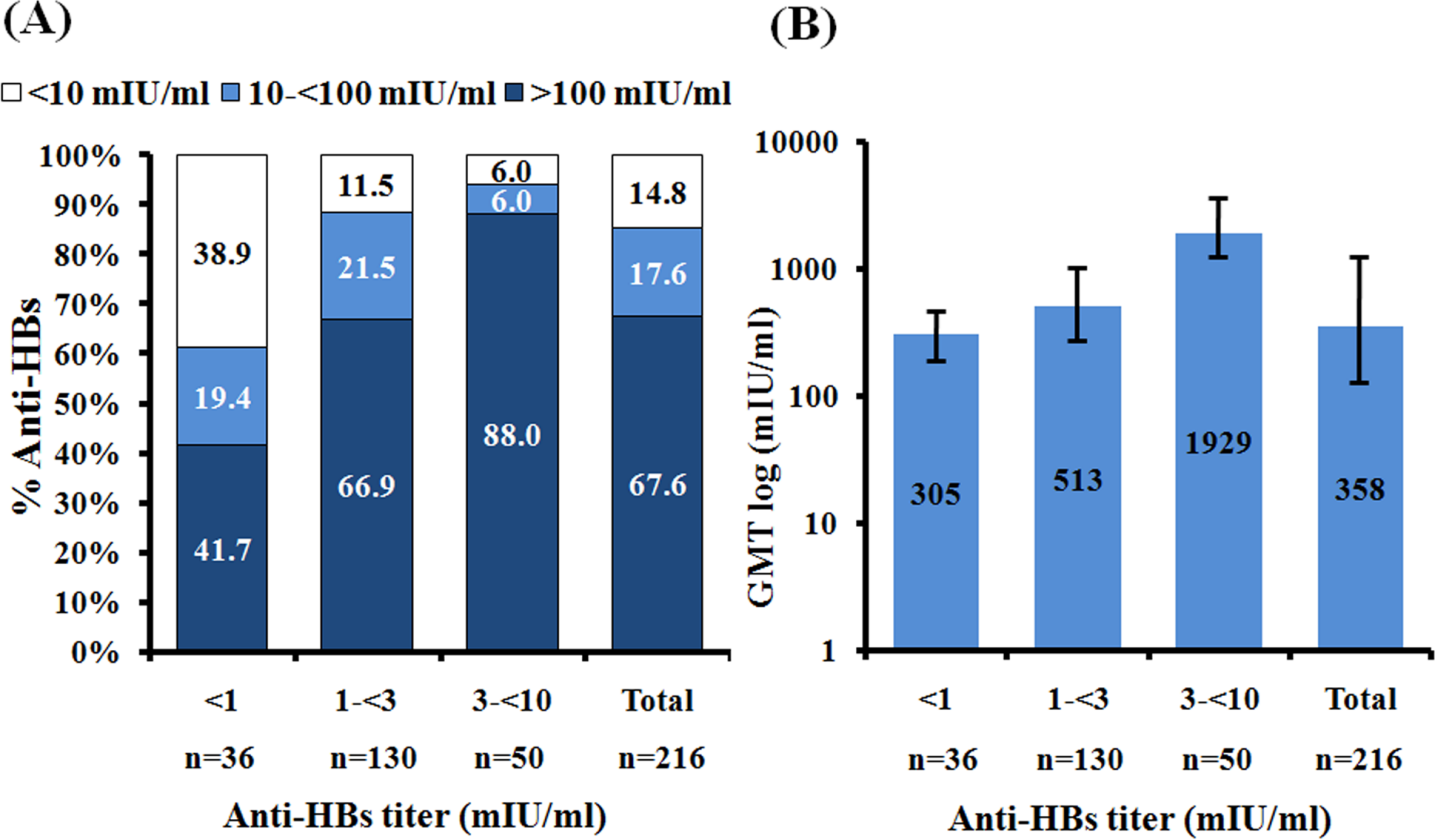
Anti-HBs levels post-booster among individuals with varying pre-existing anti-HBs. (A) Individuals were grouped based on their pre-existing anti-HBs (<1 mIU/ml, 1-<3 mIU/ml and 3-<10 mIU/ml shown on the X-axis). Percentage of individuals with post-booster anti-HBs (Y-axis) of <10 mIU/ml (white), 10-<100 mIU/ml (light blue), and >100 mIU/ml (dark blue). (B) Geometric mean titers of anti-HBs post-booster among individuals with different pre-existing anti-HBs.

The magnitude of the response to the HB vaccine booster was dependent on the pre-existing anti-HBs titers. Post-booster anti-HBs >100 mIU/ml was observed in 41.7% and 88% of individuals with pre-booster anti-HBs <1 mIU/ml and 3-<10 mIU/ml, respectively (Fig 4A). The correlation between higher pre-booster anti-HBs titers and higher post-booster response of anti-HBs ≥100 mIU/ml was further supported by the observed increasing GMT post-booster with increasing pre-booster anti-HBs status (Fig 4B). Thus, the majority of the individuals who received the HB vaccine booster showed protective anti-HBs titers.

## Discussion

The Thai Ministry of Public Health reported that HB vaccine coverage between 1996 and 2000 was approximately 94%. Our study showed that up to 93% of young adults had anti-HBs <10 mIU/ml despite more than 94% of vaccine coverage during childhood. Our data were comparable with the national survey of the HB seroprevalence among different age groups throughout Thailand [8].

Neither screening for HB immunity nor routine HB vaccine booster is currently recommended for the general population [9, 10, 11, 12]. It had been assumed that upon the completion of the primary HB vaccination series, sufficient anti-HBs were made and immune memory was established [13]. In contrast, natural HBV infection after a long incubation period of 3–6 months precedes secondary immune response [14]. Furthermore, chronic HBV infection after the primary anti-HBs response is infrequent even when the anti-HBs is <10 mIU/ml [10, 15, 16, 17]. However, some individuals such as health-care workers, transfusion patients, sex workers, men who have sex with men, inmates, and intravenous drug users may be at a greater risk of HBV infection. According to the current recommendation for health-care personnel, documented responder (anti-HBs ≥10 IU/ml) who previously completed HB vaccine series did not require a booster [10, 11, 18]. Unfortunately, childhood vaccination records in Thailand are often not well-kept, so the routine screening for anti-HBs during enrollment and catch-up vaccination are necessary to ensure protective antibody level [10].

Overall, >85% of young adults with pre-existing anti-HBs <10 mIU/ml who had received an HB booster demonstrated protective anti-HBs at 6 weeks post-vaccination. However, we found that response to the HBV booster largely depended on the pre-existing anti-HBs level. Those with pre-existing anti-HBs <1 mIU/ml were less likely to achieve protective immunity compared to anti-HBs 1-<10 mIU/ml. Thus, a complete HB vaccine series of 3 doses at 0, 1, and 6 months are highly suggested regardless of their vaccination history. Meanwhile, the data from this study suggest that the majority of young adults (88–94%) with anti-HBs 1-<10 mIU/ml who are at risk of HBV exposure will require only one HB booster to achieve protective anti-HBs.

HBV vaccine derived from plasma was first licensed in 1981 and a recombinant vaccine was registered in 1986 [19]. The vaccine proved highly effective and had been used to prevent and reduce vertical transmission from HBV carrier mothers to their newborns at >90% [5, 20]. The vaccine also has long-term protection for at least 20 years [21–27]. Even though an adult infected with HBV is at a very low risk of developing chronic HB infection (1–5%) [2], horizontal transmission from blood-borne infection in healthcare settings, including from patients with active HB, warrants maximizing protective immunity among healthcare workers.

Approximately 96% of young adults achieved seroprotection after completing three doses of HB vaccination [28]. Only 4% were unsuccessful in producing anti-HBs levels higher than the recommended protective levels. Since HBV has a long incubation time of 3–6 months [29], a booster vaccine can prevent infection because anti-HBs can be stimulated rapidly [10]. Moreover, previous studies showed that in HBeAg-positive mothers, newborns who were HB vaccinated within the first year of life remained HBsAg-negative. Only 2% of these newborns were eventually anti-HBc-positive later in life. To date, none of the vaccinated newborns is known to develope liver disease [22, 30, 31].

## Conclusion

Healthcare workers are at an increased risk from blood-borne transmission of HBV especially when anti-HBs levels are insufficiently protective (<10 mIU/ml). Selective HB booster in high-risk adolescents based on their pre-existing anti-HBs titer may provide the necessary seroconversion.

## Supporting information

S1 Table Initiate screening of medical student. (XLS)

S2 Table Anti-HBs titers of medical student after post-booster. (XLS)
